# Herbal medicine (Gancao Xiexin decoction) for Behcet disease: A systematic review protocol

**DOI:** 10.1097/MD.0000000000012324

**Published:** 2018-09-14

**Authors:** Dongjie Wu, Weiwei Lin, Ka-wang Wong

**Affiliations:** a School of Chinese Medicine, Hong Kong Baptist University, Hong Kong; b Graduate School, Zhejiang Chinese Medical University, Hangzhou; c The First Affiliated Hospital, Wenzhou Medical University, Wenzhou, Zhejiang Province; d Department of Chemistry, Hong Kong Baptist University, Hong Kong SAR, China.

**Keywords:** Behcet disease, Chinese herbal, Gancao Xiexin Decoction, medicine randomized controlled trials, systematic review

## Abstract

**Background:**

Behcet disease (BD) is a systemic vasculitis that causes blood vessel inflammation throughout body and no cure exists. The purpose of this review is to evaluate the safety and efficacy of Gancao Xiexin Decoction (GCXX) in the treatment of BD and provide a clinical reference for the treatment of the refractory disease.

**Methods:**

First, the researcher will retrieve the following database based on the established search strategy: MEDLINE (PubMed), the Cochrane Central Register of Controlled Trials (CENTRAL), EMBASE, Allied and Complementary Medicine Database (AMED), China National Knowledge Infrastructure, Wanfang, VIP (Journal Integration Platform). The quality of the literature will be evaluated according to Cochrane's handbook and review inclusion criteria. Data will be extracted and data of sufficient homogeneity was combined using Review Manager (RevMan) software V5.3.5.

**Results:**

This study will provide a high quality synthesis of herbal medicine (Gancao Xiexin decoction) for Behcet disease.

**Conclusions:**

The conclusion of our systematic review will provide evidence to judge whether herbal medicine (Gancao Xiexin decoction) for Behcet disease is an effective intervention for patient with menopausal women.

**Trial registration number:**

PROSPERO CRD 42018104099.

**Strengths and limitations of this study**

- This study is a comprehensive, objective, and normative systematic review on the efficacy and safety of Chinese herbal medicine (Gancao Xiexin Decoction) for Behcet disease.
- Study screening, data extraction, and assessment of the risk of bias will be conducted independently by 2 researchers.
- The systematic review may provide researchers more references to help relieve BD patient symptom.
- However, more high-quality randomized controlled trials
- (RCTs) following standardized guidelines are needed.

## 1 Introduction

Behcet disease (BD) characterized by ulcers in the mouth and on the genitals, also called Behcet syndrome, is a systemic vasculitis that causes blood vessel inflammation throughout body.^[1–3]^ There are some existing pharmacotherapies for treating the disease but the treatment effect is not very satisfactory,^[4–6]^ and many patients are suffering from poor treatment. One of available treatments corticosteroids can reduce severe joint pain, skin sores, eye disease during acute exacerbations, but long-term corticosteroids may cause several side effects such as diabetes, osteoporosis, and no cure exists.^[7]^ As conventional medicines for moderate and severe BD patients have been reported to be associated with unwanted side effects, many patients with BD have sought other therapies.^[8–10]^

Chinese herbal medicine (CHM) is one of the most commonly used complementary therapies with a long history of being applied for the treatment of skin disorder.^[11–13]^ It is an interesting finding that the ancient Chinese medical text Jinkui Yaolue (Essentials of the golden cabinet) written by Zhang Ji has a description of disease called “huhuo” with similar symptom to BD and gives his treatment methods. In 1964, a doctor of traditional Chinese medicine reports the discovery of the old treatment method.^[14]^ Since then, Chinese herbalists do some clinical trials and laboratory studies using improved traditional treatment based on Zhang Zhongjing.^[15]^ This review will collect these latest findings and provide evidence for the safety and effectiveness of the treatment.

Although Gancao Xiexin Decoction (GCXX) is widely used, its therapeutic effect is still controversial. In Western countries, some clinics are using this prescription to treat BD, but official journals lack coverage of this treatment.^[16]^ Thus, we search the clinical evidence for CHM (GCXX) for BD in systematic reviews and randomized controlled trials (RCTs) published from 1960 to 2018 were reviewed.

This systematic review and meta-analysis comprehensively provided objective evidence and efficacy evaluation of GCXX in the treatment of BD based on current research, providing new research ideas for the treatment of this disease. We hope that our review will help patients with this disease.

## 2 Methods

### 2.1 Criteria for considering studies for this review

#### 2.1.1 Types of studies

RCTs utilize GCXX and variant formulas will be included. There is no restriction on the language of the literature. The inclusion criteria will be as follows: RCTs of patients diagnosed with cholestasis that met the criteria of the International Classification Standard developed by the International BD Research Group in 1989. GCXX was used alone or in combination with an essential therapy, compared with the essential therapy (Western medicine or placebo) as a control. Outcome measurements will include the clinical effective rate.

#### 2.1.2 Types of participants

We will include BD patients based on international diagnostic criteria and the consensus of Chinese experts.

#### 2.1.3 Types of interventions

The interventions of GCXX and any prescription containing GCXX are eligible for the inclusion criteria.

### 2.2 Types of outcome measures

#### 2.2.1 Primary outcomes

The effectiveness rate will be the primary outcome for assessing the degree of disease recovery.

#### 2.2.2 Secondary outcomes

The erythrocyte sedimentation rate and C-reactive protein will be the secondary outcomes of the review.

### 2.3 Search methods for the identification of studies

#### 2.3.1 Electronic searches

The following electronic databases will be searched from their inception.

MEDLINE(PubMed), the Cochrane Central Register of Controlled Trials (CENTRAL), EMBASE, Allied and Complementary Medicine Database (AMED), China National Knowledge Infrastructure (CNKI), Wanfang, VIP (Journal Integration Platform).

#### 2.3.2 Other sources

We will search for documents in the following ways:

Google

Clinical trials.gov

Other related meeting materials

### 2.4 Search strategy

We will adopt a combination of keyword and free word search strategy: keyword is “Gancao Xiexin decoction,” “Behcet disease,” and “Randomized controlled trials.”

### 2.5 Data collection and analysis

#### 2.5.1 Selection of studies

**Figure 1:**
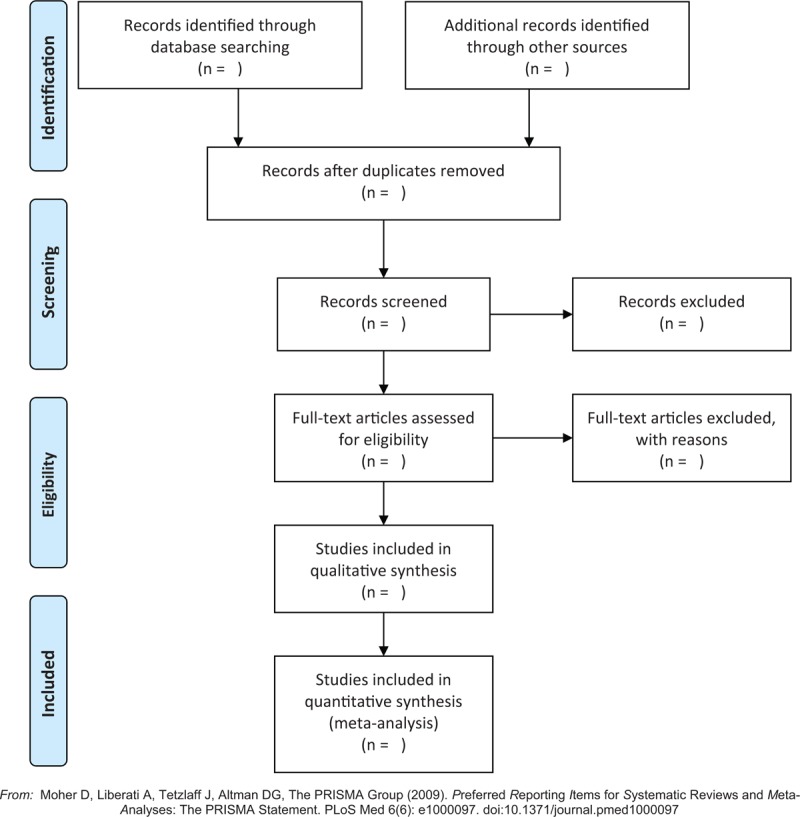
Flow diagram of study selection process.

Two researchers (DW and WL) independently scanned all articles, and data were extracted from the articles according to a standardized data extraction form (Fig. 1). Then, 2 investigators independently read the selected papers and made a final decision. Disagreements were settled through consensus.

#### 2.5.2 Data extraction and management

We use Endnote to manage search literature and remove duplicate literature. We use EXCEL to collect data, including groupings, number of samples per group, and measurements of research effects. The data extraction of DW and WL, the inability to reach an agreement is determined by the third author (K-wW).

#### 2.5.3 Assessment of risk of bias in included studies

Two researchers, according to the “risk of bias assessment tool” of the Cochrane handbook, evaluated the methodological treatment of the included literature, and the disagreement where the opinions could not be agreed was resolved by (K-wW).

##### 2.5.3.1 Measures of treatment effect

For discontinuous variables, the effect indicator is represented by the risk ratio value and 95% confidence interval (CI). For continuity variables, the effect indicator is represented by the odds ratio value and 95% CI.

#### 2.5.4 Unit of analysis issues

The unit of analysis issue appears in clinical trials with long treatment durations. For cross-over trials, we will use the data from the first phase. If there are multiple time observation points, the data will be divided into short-term (within 4 weeks) and long-term (more than 4 weeks) follow-up.

#### 2.5.5 Dealing with missing data

We will consult the author if the data lacks or other data is unclear. If the author provides sufficient information, we may conduct an Intention-to-Treat Analysis.

#### 2.5.6 Assessment of heterogeneity

We will use the I^2^ value to judge the heterogeneity. When I^2^ = 0 (if I^2^ is negative, we set it to 0), it means that no heterogeneity is observed. The larger the I^2^ value, the greater the heterogeneity; the I^2^ values 25%, 50%, and 75% indicate the low, medium, and high degrees of heterogeneity, respectively. If I^2^>50%, there is a significant heterogeneity.

#### 2.5.7 Assessment of reporting biases

If more than 10 articles are included, we will use the symmetry of the funnel plot in RevMan to determine heterogeneity, or use the Stata software to perform the Egger test.

### 2.6 Data synthesis

For the difference in efficacy between the intervention group and the treatment group, we will use the risk ratio value and 95% CI to illustrate. Researchers will evaluate the heterogeneity of the included literatures according to the *χ*^2^ and the I^2^ tests. Most of the statistics will be counted using Review Manager (RevMan), V.5.3 software provided by Cochrane Collaboration's software programme.

### 2.7 Subgroup analysis and investigation of heterogeneity

If the heterogeneity is large, we will use a subgroup analysis to reduce heterogeneity.

### 2.8 Sensitivity analysis

We will conduct sensitivity analysis as much as possible to explore the extent to which a single study affects the combined effect and the robustness of the results. If there is a big change in the results after exclusion, the sensitivity is lower and the results are more stable and credible. On the contrary, if the difference is greater or even the opposite conclusion after the exclusion, the sensitivity is higher and the result is less robust. It should be very cautious in interpreting the results and conclusions.

### 2.9 Assessment of quality of evidence

We will use GRADE (The Grading of Recommendations Assessment Development and Evaluation) to assess the quality of systematic review.

### 2.10 Ethics and dissemination

There is no special ethical statement that needs to be announced, the proposal is for systematic review. This review will be published and disseminated after peer review, and will be used for conference presentations.

## 3 Discussion

There are 3 deficiencies in the study. First, the literature on GCXX only records the symptoms similar to BD, but it is difficult to ascertain that the disease golden chamber recorded is BD discovered in modern times. Second, the GCXX has a large difference with some deformations, and the treatment of traditional Chinese medicine needs to be based on Traditional Chinese medicine syndromes.^[17,18]^ Third, the design of the randomized clinical trial is not sufficiently standardized. We will assess confidence in the evidence using GRADE criteria. Although there are many deficiencies, this treatment method still has value in the current difficult development of BD. The Chinese medicine prescription still has controversy about its effectiveness treating BD, and our job is to make its treatment effect more systematic. For determining the therapeutic effect of GCXX, more high-quality clinical evidences are needed.

## Author contributions

Contributors: DJW and WWL contributed to the conception of the study. The manuscript protocol was drafted by DJW and was revised by KWW. The search strategy was developed by all the authors and will be performed by DJW and WWL, who will also independently screen the potential studies, extract data from the included studies, assess the risk of bias and complete the data synthesis. KWW will arbitrate in cases of disagreement and ensure the absence of errors. All authors approved the publication of the protocol.

**Methodology:** Weiwei Lin.

**Writing – original draft:** Dongjie Wu.

**Writing – review & editing:** Dongjie Wu, Ka-wang Wong.

Dongjie Wu orcid: 0000-0001-5760-5615
